# Evaluation of the dosimetry approaches in ablation treatment of thyroid cancer

**DOI:** 10.1002/acm2.12350

**Published:** 2018-06-01

**Authors:** Nalan Alan Selcuk, Turkay Toklu, Seval Beykan, Serife Ipek Karaaslan

**Affiliations:** ^1^ Department of Nuclear Medicine Yeditepe University Hospital Istanbul Turkey; ^2^ Department of Physics Yeditepe University Istanbul Turkey; ^3^ Department of Nuclear Medicine University of Würzburg Würzburg Germany

**Keywords:** bone marrow dosimetry, remnant tissue dosimetry, thyroid ablation treatment

## Abstract

In this study, we aimed to evaluate dosimetric approaches in ablation treatment of Differentiated Thyroid Carcinoma (DTC) without interrupting the clinical routine. Prior to therapy, 10.7 MBq ^131^I in average was orally given to 24 patients suffering from DTC. MIRD formalism was used for dosimetric calculations. For blood and bone marrow dosimetry, blood samples and whole‐body counts were collected at 2, 24, 72, and 120 h after I‐131 administration. For remnant tissue dosimetry, uptake measurements were performed at the same time intervals. To estimate the remnant volume, anterior and lateral planar gamma camera images were acquired with a reference source within the field of view at 24 h after I‐131 administration. Ultrasound imaging was also performed. Treatment activities determined with the fixed activity method were administered to the patients. Secondary cancer risk relative to applied therapy was evaluated for dosimetric approaches. The average dose to blood and bone marrow were determined as 0.15 ± 0.04 and 0.11 ± 0.04 Gy/GBq, respectively. The average remnant tissue dose was 0.58 ± 0.52 Gy/MBq and the corresponding required activity to ablate the remnant was approximately 1.3 GBq of ^131^I. A strong correlation between 24th‐hour uptake and time‐integrated activity coefficient values was obtained. Compared to fixed activity method, approximately five times higher secondary cancer risk was determined in bone marrow dosimetry, while the risk was about three times lower in lesion‐based dosimetry.

## 1 INTRODUCTION

Patients with well‐differentiated thyroid cancer have a long life expectancy after successful therapy; even though a lifelong risk of recurrence such as local, regional, or less commonly distant metastases may develop.^1^ Most of these recurrences can be overcome successfully with radioiodine treatment after surgery; for this reason, ablation of remnant thyroid tissue is critical.

Radioactive iodine ablation (RAI) treatment has been used for most of the cases after surgery for almost 60 yr.^1^ RAI treatment is usually preferred to extinguish the remnant tissue in any location. This treatment also provides an increase in sensitivity of imaging methods and thyroglobulin (Tg), a specific tumor marker. There are clinical studies showing that RAI treatment reduces the risk of tumor recurrence, metastases, and long‐term mortality. The treatment activity is generally decided according to the patients' clinical situation in most of nuclear medicine clinics. Suggested treatment activity for the ablation treatment varies from 0.9 to 7.4 GBq in the literature.^2^, ^3^, ^4^, ^5^, ^6^ Recurrence ratios in the studies do not illustrate a significant difference in the patients who are applied 1.1–1.9 GBq RAI treatment and who are applied 1.9–7.4 GBq (7% and 9%, respectively).^1^ One of the randomized prospective study shows that 81% of the patients have been ablated in their first treatment with 1.1 GBq RAI and this ratio is 84% with 3.7 GBq.^7^ Hence, there is no certain treatment activity decided in the RAI treatment to ablate the remnant tissue based on the fixed activity method.

Nowadays, the required ablation treatment activity for the patient is determined according to the fixed activity method, which is based on clinical and pathological observations and dosimetry methods as well. Two dosimetry approaches are used for the RAI treatment.^8^ The first one, defined by Benua et al.^9^ in 1962, is to give maximum safe activity to patients. As stated in their study, they aimed to give maximum activity without exceeding 2 Gy dose to the blood as a surrogate for the bone marrow, the critical organ in RAI treatment. The second dosimetry technique is lesion‐based dosimetry, which was first mentioned in the study of Maxon et al. in 1983.^10^ In their work, they suggested that minimum 300 Gy to the remnant thyroid tissue and minimum 80 Gy to metastases should be given for a successful treatment. The most important advantage of this method was to ablate the remnant tissue with lower activities.

This study aimed to determine the absorbed dose to bone marrow and remnant thyroid tissue of the patients with Differentiated Thyroid Carcinoma before the ablation therapy without interrupting the clinical routine. The associated relative secondary cancer risk was also analyzed.

## 2 MATERIALS AND METHODS

Twenty‐four patients (F/M: 18/6, average age: 40.8 ± 12.2, TSH: 80.3 ± 33.3, and Tg: 9.2 ± 8.9) suffering from papillary and follicular thyroid carcinoma were included in this study. All patients underwent a routine uptake study before ablation treatment. For this purpose, the average of 10.8 ± 1.7 MBq ^131^I was orally given to the patients. The administered activities for each patient can be seen in Table 1. Blood samples, anterior and posterior whole‐body counts, Ultrasound and planar gamma camera images were used for dosimetry calculations.

**Table 1:** Patients' data

Patient No	Gender	Age	TSH (*μ*IU/ml)	Tg (ng/ml)	Uptake study activity (MBq)	Ablation treatment activity (GBq)
1	F	43	74	9	11.1	3.7
2	F	45	49	14	9.4	3.7
3	F	16	137	<0.001	9.3	3.7
4	F	52	52	13	11.6	3.7
5^a^	F	11	96	4	8.8	1.7
6	F	47	82	29	11.1	3.7
7	M	42	15	31	10.2	3.7
8	M	42	39	0.1	9.9	3.7
9	F	40	52	0.4	11.2	3.7
10	M	33	56	23	11.6	5.6
11	F	40	113	17	11.8	3.7
12	F	53	48	1	10.5	3.7
13	F	36	70	6	10.8	3.7
14	F	57	113	9	11.3	5.6
15	M	42	104	3	10.8	3.7
16	F	51	99	8	10.8	5.6
17	F	50	84	10	10.8	3.7
18	F	49	100	0.2	11.2	3.7
19	F	37	150	5.9	9.5	3.7
20	M	65	127	2.3	18.1	3.7
21	F	28	77	3.3	10.5	3.7
22	F	28	74	0.3	9.3	3.7
23	F	28	87	15.0	9.5	3.7
24	M	44	32	17.9	9.9	3.7
Average:		40.8	80.3	9.2	10.8	3.8
Standard deviation:		12.2	33.3	8.9	1.7	0.8

a Pediatric patient.

Blood, bone marrow, and remnant thyroid tissue doses were calculated for each patient using MIRD scheme.^11^

### 2.A Blood and bone marrow dosimetry

EANM blood and bone marrow dosimetry in differentiated thyroid cancer therapy guideline^12^ was used to calculate blood and bone marrow doses. According to the guideline, the average dose to blood can be calculated by:

(1) $$\frac{{\overset{¯}{D}}_{\mathrm{blood}}}{A_{0}}\left[\frac{\text{Gy}}{\text{GBq}}\right]=108\times \tau_{\mathrm{millilitre}\;\mathrm{of}\;\mathrm{blood}}\left[h\right]+\frac{0.0188}{{\left(\mathrm{wt}[\mathrm{kg}]\right)}^{2/3}}\times \tau_{\mathrm{total}\;\mathrm{body}}\left[h\right]$$

where *A*_*0*_ is the administered activity, *τ* is the time‐integrated activity coefficient (TIAC) (formerly known as residence time), and *wt* is patient's body weight. Bone marrow dose calculation equation given in the guideline is:

(2) $$\frac{D_{\mathrm{redmarrow}}}{A_{0}}\left[\frac{\text{Gy}}{\mathrm{GBq}}\right]=61\times \tau_{\mathrm{millilitre}\;\mathrm{of}\;\mathrm{blood}}\left[h\right]+\frac{0.106}{\mathrm{wt}[\mathrm{kg}]}\times \tau_{\mathrm{total}\;\mathrm{body}}\left[h\right]$$

To calculate the activity in the blood, 2 ml blood samples at 2, 24, 72, and 120 h were analyzed using the calibrated well‐type NaI gamma counter (Capintec Captus‐3000, Capintec, Inc. NJ, USA).

Anterior and posterior whole‐body counts at 2, 24, 72, and 120 h were measured using the NaI probe detector (Capintec Captus‐3000, Capintec, Inc. NJ, USA) at a distance of 2 m from the patient. The probe was positioned at the bottom level of the patient's sternum.^13^ The position of the probe and the patient was constant for the counting repeatability.

Whole‐body counts at 2 h were taken as a reference that represent the total administered activity because the patients were not allowed micturition until the measurements were performed. Position changes of the activity in the body were ignored.

The TIACs were calculated using the software solution NUKFIT,^14^ choosing the optimal fit functions as proposed by the code. A systematic error in activity determination of 10% was assumed in the calculations.

Another bone marrow dose calculation was performed according to the EANM bone marrow dosimetry guideline (EANM‐2010).^15^ For thyroid treatment with ^131^I‐NaI, only the dose from extracellular fluid to bone marrow and dose from the rest of the body to bone marrow were calculated as mentioned in the guideline. To estimate activity concentration in bone marrow based on activity concentration in blood, red marrow‐to‐blood activity concentration ratio (RMBLR) was used as unity.^15^ S factor for the rest of the body was calculated according to EANM‐2010.^15^ Cristy and Eckerman's^16^ 10‐year‐old child, adult female and male MIRD phantoms were used. S factors for bone marrow to bone marrow and whole‐body to bone marrow were taken from the study by Stabin and Siegel.^17^

### 2.B Remnant thyroid tissue dose calculation

For the calculation of dose to remnant thyroid tissue, the Unit‐density Sphere Model was used.^18^ In this model, the tissues were simulated as spheres with density of 1 g/cm^3^.

The remnant tissue volumes were determined using both Ultrasound (US) and planar gamma camera images taken at 24 h after ^131^I intake. In US imaging, the diameters of the remnant tissue were measured in three dimensions. In the gamma camera imaging, a cylindrical source (having a diameter of 2.5 cm, height of 1 cm, filled with 370 kBq of ^131^I) was located at the same distance with the thyroid. The diameters of the remnant tissue were determined by the comparison of the full width at half maximums (FWHMs) of the profiles drawn on the center of both the cylindrical source and the remnant.

To calculate the cumulative activity in the thyroid, uptake values at 2, 24, 72, and 120 h after injection were determined using the thyroid uptake system. The time–activity curve of the thyroid was integrated using a trapezoidal integration. After the last time point, only the physical decay was taken into account.

The S factors for the remnant tissue dosimetry were determined from the fit function of the data by Stabin and Konijnenberg.^18^

### 2.C Relative secondary cancer risk assessment in dosimetry approaches

Relative secondary cancer risk assessments in dosimetry approaches were done considering that doses to organs were directly proportional to the administered activity, and relative secondary cancer risk was directly proportional to the organ doses within the same patient. Thus, relative secondary cancer risk in dosimetry approaches was determined by dividing activity calculated in the dosimetry approach by the applied treatment activity.

## 3 RESULTS

All patients were in stage 1 according to TNM.^19^ Seventeen of the patients had papillary thyroid cancer and one of them minimal invasive follicular cancer. Radioactivity of 3.7 GBq ^131^I was administered to 20 patients, while three of the patients were administered 5.6 GBq ^131^I because of having lymph node invasion. For the pediatric patient (patient no:5 in Table 1) 1.7 GBq ^131^I was administered according to her mass index. Re‐operation was suggested for the patients with 24th‐hour uptake value higher that 15% (patients 7, 17, 23, and 24). These patients could not be operated due to the existence of thyroiditis.

Blood and bone marrow doses according to the two different EANM guidelines are given in Table 2. There was no significant difference observed between bone marrow doses according to two EANM guidelines (*P* > 0.05). Significant differences were found between blood and bone marrow doses from both guidelines (*P* < 0.05).

**Table 2:** Blood and bone marrow doses according to the two different EANM guidelines

Patient no.	Blood dose from EANM‐2008^(12)^ (Gy/GBq)	Bone marrow dose from EANM‐2008^(12)^ (Gy/GBq)	Bone marrow dose from EANM‐2010^(15)^ (Gy/GBq)
1	0.15	0.12	0.11
2	0.17	0.15	0.14
3	0.17	0.12	0.11
4	0.16	0.14	0.13
5	0.19	0.15	0.14
6	0.12	0.10	0.09
7	0.20	0.16	0.15
8	0.23	0.18	0.17
9	0.10	0.08	0.07
10	0.07	0.06	0.05
11	0.20	0.15	0.14
12	0.18	0.13	0.12
13	0.09	0.07	0.07
14	0.16	0.11	0.11
15	0.15	0.11	0.10
16	0.10	0.07	0.07
17	0.19	0.15	0.14
18	0.09	0.07	0.07
19	0.13	0.11	0.10
20	0.14	0.07	0.10
21	0.10	0.05	0.08
22	0.09	0.04	0.07
23	0.16	0.08	0.14
24	0.14	0.07	0.12
Average:	0.15	0.11	0.11
Standard deviation:	0.04	0.04	0.03

Effective half‐life of the activity was determined as 12.9 h for the total body and 9.6 h for the blood. The corresponding TIAC values were 39.1 and 4.0 h, respectively.

As for thyroid remnant tissue, 24th‐hour uptake, effective half‐life (T_½_), and TIAC values are shown in Table 3.

**Table 3:** Twenty‐forth‐hour uptake, effective half‐life (T_1/2_), and time‐integrated activity coefficient (TIAC) values in remnant thyroid tissue

Patient no.	24th hour uptake (%)	Thyroid	
		T_½_ (h)	TIAC (h)
1	10.1	203.7	29.0
2	8.3	255.7	26.0
3	4.7	70.2	6.8
4	10.7	222.5	26.6
5	2.6	104.3	5.5
6	4.8	155.5	9.6
7	16.7	297.8	64.2
8	12.0	216.9	35.7
9	1.0	82.4	2.4
10	2.1	126.8	5.0
11	5.4	75.8	10.2
12	6.8	229.5	21.4
13	1.9	275.1	6.6
14	1.6	224.5	4.2
15	1.8	107.6	3.2
16	1.3	76.9	3.0
17	15.5	198.1	41.8
18	5.9	234.1	18.4
19	7.1	285.0	25.7
20	1.7	242.0	5.5
21	2.1	242.0	5.6
22	7.3	326.7	29.3
23	19.6	246.2	62.7
24	16.5	300.7	61.4
Average:	7.0	200.0	21.3
Standard deviation:	5.5	78.3	19.4

Thyroid tissue volumes measured both with gamma camera and US and their related doses are indicated in Table 4. The volume values were in a wide range between two modalities (average 4.6 cm^3^ with gamma camera, while 1.4 cm^3^ with US).

**Table 4:** Thyroid tissue volumes with gamma camera and US and related doses

Patient no	Gamma camera			US		
	Volume (cm^3^)	Remnant thyroid tissue dose (Gy/MBq)	Required activity for 300 Gy to Remnant (GBq)	Volume (cm^3^)	Remnant thyroid tissue dose (Gy/MBq)	Required activity for 300 Gy to Remnant (GBq)
1	8.7	0.40	0.75	1.1	2.94	0.10
2	5.0	0.61	0.49	0.2	15.35	0.02
3	6.6	0.12	2.44	1.9	0.40	0.75
4	6.3	0.51	0.59	0.8	3.61	0.08
5	2.6	0.25	1.22	0.2	3.90	0.08
6	5.7	0.20	1.49	1.0	1.08	0.28
7	9.3	0.83	0.36	3.1	2.38	0.13
8	4.4	0.96	0.31	1.3	3.22	0.09
9	2.5	0.11	2.71	0.5	0.49	0.61
10	3.7	0.16	1.90	0.5	1.08	0.28
11	1.6	0.71	0.42	1.0	1.16	0.26
12	7.4	0.34	0.87	2.3	1.06	0.28
13	1.9	0.40	0.75	5.5	0.14	2.10
14	6.1	0.08	3.66	2.6	0.19	1.57
15	5.5	0.07	4.36	0.1	3.35	0.09
16	5.3	0.07	4.47	0.2	1.77	0.17
17	4.7	1.04	0.29	0.9	5.42	0.06
18	2.2	0.97	0.31	1.0	2.08	0.14
19	4.3	0.71	0.42	0.7	3.91	0.08
20	1.9	0.34	0.89	0.8	0.81	0.37
21	3.5	0.19	1.61	3.8	0.17	1.72
22	2.9	1.18	0.25	0.9	3.84	0.08
23	3.3	2.20	0.14	1.1	6.70	0.04
24	4.8	1.51	0.20	1.5	4.77	0.06
Average:	4.6	0.58	1.29	1.4	2.91	0.39
Standard deviation:	2.1	0.52	1.29	1.3	3.14	0.56

**Figure 1:**
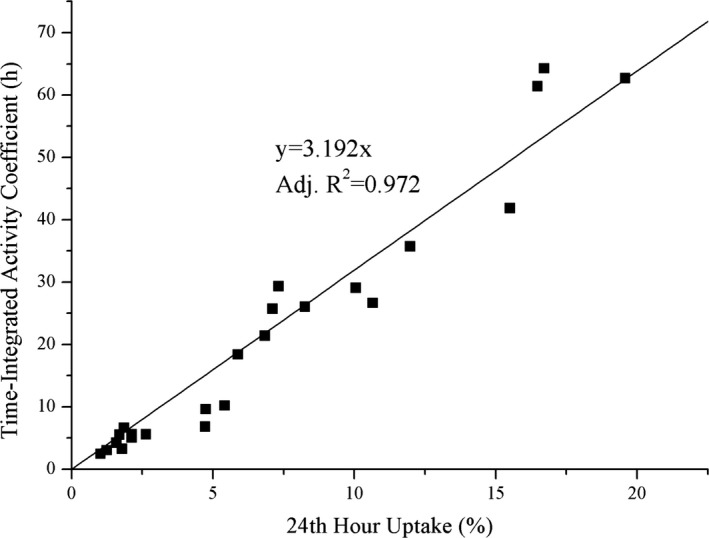
The relationship between 24th‐hour uptake value and TIAC.

To be used in dosimetry calculations for future patients, the correlation between 24‐h uptake and TIAC values was also investigated. A strong correlation was obtained between these quantities (Adjusted *R* ^2^ = 0.972) (Fig. 1).

Relative secondary cancer risk in selected dosimetry approaches with respect to the applied therapy is given in Table 5.

**Table 5:** Relative secondary cancer risk in dosimetry approaches relative to applied therapy

Patient no.	Ablation treatment activity (GBq)	Relative secondary cancer risk		
		Blood dosimetry (EANM‐2008^(12)^)	Bone marrow dosimetry (EANM‐2010^(15)^)	Remnant thyroid tissue dosimetry (gamma camera)
1	3.7	3.7	4.8	0.2
2	3.7	3.2	4.0	0.1
3	3.7	3.1	4.8	0.7
4	3.7	3.5	4.1	0.2
5	1.7	6.3	8.9	0.7
6	3.7	4.4	5.9	0.4
7	3.7	2.8	3.5	0.1
8	3.7	2.3	3.2	0.1
9	3.7	5.2	7.7	0.7
10	5.6	4.8	6.6	0.3
11	3.7	2.7	3.9	0.1
12	3.7	3.0	4.3	0.2
13	3.7	6.0	7.9	0.2
14	5.6	2.2	3.4	0.7
15	3.7	3.5	5.4	1.2
16	5.6	3.4	5.1	0.8
17	3.7	2.9	3.9	0.1
18	3.7	6.0	8.2	0.1
19	3.7	4.1	5.3	0.1
20	3.7	3.9	5.4	0.2
21	3.7	5.4	7.2	0.4
22	3.7	6.1	7.4	0.1
23	3.7	3.3	3.8	0.0
24	3.7	3.9	4.5	0.1
Average:		4.0	5.4	0.3
Standard deviation:		1.2	1.7	0.3

## 4 DISCUSSION

Remnant tissue ablation has been well established and accepted in the management protocol of DTC for many years. The National Thyroid Cancer Treatment Cooperative Study group had confirmed that postoperative RAI treatment was associated with improved cancer‐specific mortality rates and reduced disease progression in well DTC.^20^

With this study, it is proven that remnant ablation can be achieved by either administering an empiric fixed activity of ^131^I or using dosimetry‐guided techniques. Because of the technical and logistic difficulties, many clinics have preferred to use the fixed activity technique with 0.9–7.4 GBq of ^131^I.^2^, ^3^, ^4^, ^5^, ^6^ However, the activity for ablation of the remnant tissue is an ongoing debate. The administrated activity decided regardless of the bio‐kinetics of iodine is the main disadvantage of fix activity treatment method. This drawback may cause over or insufficient treatment. For this reason, dosimetry approaches increasingly gain importance.

Although generally it is used for the patients with metastatic thyroid cancer, maximum safe activity calculation was also included in this study. The main reason for this is to be aware of the safe activity levels used in the treatments. Besides, not only blood doses for which Benua et al.^9^ found a maximum safe absorbed dose of 2 Gy, but also bone marrow doses, for which an absorbed dose of 2 Gy is generally considered as safe, were calculated.

Two different guidelines of EANM (EANM 2008^12^ and EANM 2010^15^) were used for bone marrow calculation, while EANM 2008 guideline^12^ was used for blood dose calculation. When compared with blood doses, calculated bone marrow doses based on EANM guidelines of 2008 and 2010 were lower by about 30%. This suggests that the activities calculated for maximum safe blood dose are also safe for bone marrow. According to the two different EANM guidelines, the average bone marrow dose values were almost the same (0.105 Gy/GBq for EANM 2008 and 0.108 Gy/GBq for EANM 2010), although there were some differences for some individual patients.

The average blood dose was found as 0.15 Gy/GBq in this study. Our result is compatible with the literature where the mean blood dose values are reported between 0.08 Gy/GBq and 0.23 Gy/GBq.^21^, ^22^, ^23^, ^24^, ^25^ Calculated maximum safe activities using both guidelines were much higher than the treatment activities decided by the fixed activity method. In other words, the bone marrow doses of the patients included in this study are much lower than the toxic dose for the bone marrow. Our findings also showed that bone marrow dosimetry is not an optimized approach for ablation treatment of thyroid cancer.

As for thyroid remnant tissue dosimetry, doses were calculated according to the tissue volumes obtained from not only gamma camera but also from US images. Remnant thyroid tissue doses based on gamma camera and US imaging measurement had an average value of 0.58 ± 0.52 and 2.91 ± 3.14 Gy/MBq, respectively (Table 4). The difference as a factor of 5 between calculations was caused by the volume measurements of the remnant tissue. Determining the remnant volume with US or computed tomography (CT) is unreliable after surgery for ablation treatment.^8^ The detection ability of the US imaging for small objects is limited. Thus, the remnant volumes determined by US images were considered as underestimating the remnant volume. The gamma camera volume estimation, however, was overestimating the remnant volume due to the spill‐out effect. Overestimating the volume leads to underestimation of the dose to remnant per unit activity. This approach gives additional safety margin for the therapies. Considering overestimated remnant thyroid tissue volume with gamma camera and 300 Gy to thyroid remnants is enough for the ablation as mentioned by Maxon et al.,^10^ approximately 1.3 GBq of ^131^I in average should be sufficient for a successful ablation treatment. A strong correlation was obtained between TIAC and 24th‐hour uptake values (Adjusted *R* ^2^ = 0.972) (Fig. 1). The correlation can be used for the estimation of TIAC values from single uptake measurement instead of a number of sequential measurements. This may increase patient comfort and prevent delays in therapy applications.

Relative secondary cancer risk assessments were performed with the assumptions stated in the Materials and Methods section. According to this, risk is approximately four‐ and fivefold higher, respectively, in either blood or bone marrow dosimetry relative to the applied therapy. Our results also suggest that the risk can be reduced to approximately one third of the applied therapy by performing lesion‐based dosimetry. As the survival rates after well‐differentiated thyroid cancer are high, secondary cancer risk due to the radioiodine therapy is an important concern. For this reason, performing remnant tissue dosimetry for ablation treatment of thyroid cancer is strongly recommended.

## 5 CONCLUSION

In the study, the dosimetry approaches in ablation treatment of well‐differentiated thyroid cancer were evaluated. It is shown that activities determined based on fixed activity treatment method can be safely given without reaching the toxic dose for bone marrow. Administration of maximum safe activities according to the bone marrow dosimetry is not recommended for ablation treatment. On the other hand, with the lesion‐based dosimetry, lower activities compared to fixed activity treatment method can be given to ablate the remnant tissue successfully. In addition to this, applying therapy according to lesion‐based dosimetry with lower activities decrease the relative secondary cancer risk approximately three times. Large dose differences between patients show the necessity of patient‐specific dosimetry.

## CONFLICT OF INTEREST

No conflicts of interest.
